# Bridging Material Variability and Tablet Performance: Optimization of Direct Compression Using Tensile Strength–Ejection Stress Mapping

**DOI:** 10.3390/pharmaceutics18030357

**Published:** 2026-03-13

**Authors:** Tibor Casian, Sonia Iurian, Alexandru Gâvan, Oana Negoi, Damaris Marusca, Adriana Marina, Maria Suciu, Dana Muntean, Alina Porfire, Anca Lucia Pop, Simona Crișan, Dumitru Cauni, Ioan Tomuță

**Affiliations:** 1 Department of Pharmaceutical Technology and Biopharmacy, Faculty of Pharmacy, “Iuliu Hatieganu” University of Medicine and Pharmacy, 400012 Cluj-Napoca, Romania; casian.tibor@umfcluj.ro (T.C.); negoi.oana@elearn.umfcluj.ro (O.N.); damarismarusca@gmail.com (D.M.); marinaadriana_22@yahoo.com (A.M.); dana.muntean@umfcluj.ro (D.M.); aporfire@umfcluj.ro (A.P.); tomutaioan@umfcluj.ro (I.T.); 2 Department of Medical Devices, “Iuliu Hatieganu” University of Medicine and Pharmacy, 4 Louis Paster, 400349 Cluj-Napoca, Romania; gavan.alexandru@umfcluj.ro; 3 National Institute for Research and Development of Isotopic and Molecular Technologies, Donath 67-103 Str., 400293 Cluj-Napoca, Romania; maria.suciu@itim-cj.ro; 4 Department of Clinical Laboratory, Faculty of Pharmacy, “Carol Davila” University of Medicine and Pharmacy, 050474 Bucharest, Romania; anca.pop@umfcd.ro; 5 RD Center, AC HELCOR, 430092 Baia Mare, Romania; simonacrisan@achelcor.ro (S.C.); dumitru.cauni@achelcor.ro (D.C.)

**Keywords:** critical material attributes, tableting, ejection stress, compaction analysis, design of experiments

## Abstract

**Objectives**: The current study presents a sequential strategy for the development of directly compressible powder formulations relying on Design of Experiments (DoE) and Compactibility-Ejection stress plots. **Methods**: Compression analysis was used to evaluate the impact of changing the sort of microcrystalline cellulose (MCC), dicalcium phosphate (DCP), the diluent ratio, lubricant type, and the inclusion of an API from different suppliers. **Results**: The effect of DCP particle size on the ejection stress was efficiently mitigated in the placebo formulations by lubrication. However, the initial differentiation between sorts was highlighted at a smaller scale when the active pharmaceutical ingredient (API) was included in the formulation. For MCC, the tensile strength was positively correlated with the level of plasticity and tabletability capacity of different sorts. The particle size was a critical attribute for the API, influencing the detachment and ejection stress values. Fine particles (d50 = 30 µm) presented increasing stress values once the compression force rose, while for coarser particles (d50 = 50 µm) these effects were limited. **Conclusions**: Material-related variability must be understood to design products and processes with adequate performance. The proposed strategy enables early identification of critical material attributes, supporting rational formulation and supplier selection to ensure consistent quality during manufacturing.

## 1. Introduction

Oral drug therapy, and especially tablets as dosage forms, are preferred by patients and drug manufacturers alike. Patients value their easy administration, while the pharmaceutical industry aims for high productivity, robust technological processes, and low costs [1,2,3]. Tablet preparation involves the compression of several components, from 4 to 12, active pharmaceutical ingredients (APIs), and excipients with different functions, such as volume increase, API release modulation, and processability improvement [2,4]. Traditionally, the selection of excipients and of their ratios was done mostly empirically, based on a few experimental results and prior knowledge of the formulators [2,5]. Currently, the concept of Quality by Design (QbD) described in International Conference of Harmonization Guidelines Q8 (R2), Q9 aims to design medicines with embedded quality assurance strategies through the rigorous knowledge of the relations between their critical quality attributes, the critical material characteristics and process parameters [6,7]. Supporting the QbD approach and aiming to overcome empirical formulation practices, expert systems like SeDeM have been developed to assess powder suitability for direct compression by integrating multiple physical parameters into a unique compression index [8]. SeDeM diagrams also enabled the identification of formulation deficiencies and the rational selection of corrective excipients [9].

Among the tablet preparation methods, direct compression is always the first choice of any formulation team, due to its straightforward nature involving only two main unit operations that ensure both cost and time effectiveness [10]. Despite its evident advantages, designing directly compressed tablets is not an easy task, as it requires appropriate powder flow, compressibility, compactibility, blend homogeneity, and it was reported that less than 20% of the APIs comply [11,12]. As in direct compression APIs and excipients only go through a mixing stage before tableting, their physical properties have an important impact on the blend flowability and compression behavior [10,13]. The Manufacturing Classification System (MCS) formalizes the link between API material properties and processing route, ranking direct compression as the simplest, yet the most property-sensitive manufacturing approach, compared to granulation methods that can accommodate broader ranges of API characteristics [14]. Particle sizes and shapes were shown to impact powder flow and, consequently, particle rearrangement during compression, with better rearrangement requiring lower compression forces for polydisperse samples [15]. Analysis of commercial products confirmed that small particle sizes and high API load are associated with more complex manufacturing routes [16]. API particle size reduction was found to increase resistance against compressibility and compactibility due to changes in deformation behavior and less fragmentation tendency [5,17]. For some materials, it had a positive impact on the mechanical strength of tablets due to extended specific surface areas but displayed a negative influence on flowability [4,5]. However, particle properties cannot be separated from the deformation behavior of each of the components of a powder blend, as fragmentation occurred at low compression pressures and depended both on the initial particle size and on the predominant deformation mechanism [18]. For example, high initial particle sizes of 355–500 µm of microcrystalline cellulose (MCC), a predominantly plastic material, tended to remain large upon compression with limited fragmentation, whereas calcium hydrogen phosphate (DCP), known as a brittle solid, underwent extensive fracturing, independent of the initial particle sizes [17]. The selection of process parameters must also be well thought-out, as they can be held accountable for both processing errors and for the correction of formulation deficiencies [19,20]. The tablet microstructure is therefore a result of the interplay of material characteristics and process parameters applied in tablet manufacturing, and was shown to impact quality attributes such as disintegration and drug release [18,21]. Along with product quality, Osamura et al. considered ejection stress as a measure that relates to the preparation performance and more precisely to the ease of tablet ejection [22].

Although tablet compression abounds in research and the understanding of the phenomena has increased over time, the development of a new product imposes numerous challenges. As described above, the performance of a process and the quality of products are influenced by a large number of factors, so most of the compression studies try to eliminate particular sources of variability to better understand the influences of certain factors, working on individual APIs or excipients [5,23], sometimes of narrow size ranges [24,25], particular shapes [25] or on binary mixtures [1]. Recent advances in tabletability modeling, such as the Vreeman-Sun equation, allowed the quantitative estimation of tensile strength as a function of compaction pressure through material-related parameters. This predictive approach for binary mixtures paves the way for the digital design of tablets [26]. While such results greatly contribute to the knowledge of powder tabletability, drug development teams still need science-based and easy-to-implement strategies for the overall understanding of their real, often complex formulations.

Therefore, the current study proposes a sequential strategy based on two QbD tools, Design of Experiments (DoE) and Multivariate Data Analysis (MVDA), to design a robust powder blend formulation for direct compression. As manufacturing companies often opt for dual supply policies to overcome unexpected API or excipient shortages [27], the study aimed to evaluate the impact of various raw material sorts and the effect of alternating suppliers on the compaction behavior. An investigation that includes a large number of input variables requires many experiments; hence, an efficient procedure to obtain product-related knowledge was needed. Compaction simulation enables the evaluation of powder tableting performance in a timely manner and with little material investment [28,29,30]. It was successfully used to support scale-up strategies and predict formulation behavior under controlled compaction conditions [31]. However, several authors have shown that compaction simulators do not fully reproduce all sub-processes encountered in rotary tablet presses, such as die filling or feeding dynamics; therefore, the direct transfer of results to industrial manufacturing requires careful consideration [32,33,34]. Nonetheless, previous results showed that the careful control of the compression analysis enables good correlations between small-scale and industrial performance under specific conditions, which underpins our choice to perform in-die and out-of-die measurements in this study [35].

Working with qualitative input variables simplifies the screening procedures associated with the development of new formulations. However, once inter-sort/-supplier differences are identified for a certain formulation component, it is recommended to identify the relevant physical properties that influence the product’s performance. In this respect, multivariate methods, i.e., Principal Component Analysis (PCA), can be efficiently used to highlight similarities and differences between excipients, considering the multiple variables collected for fundamental analysis. For this directly compressible powder formulation, each excipient was systematically characterized with respect to particle size, particle shape, flowability, and compression properties in order to better understand deformation mechanisms and predict overall blend behavior.

The proposed strategy consists of three stages: an initial screening study conducted on placebo formulations, where a high number of excipient sorts were evaluated for their compaction properties. The previously screened excipients were carried on to a second optimization study meant to reveal the influence of the API sort, with known physical and compression characteristics, as an extra qualitative variable. Finally, ejection stress assessment and extended root cause analysis using multivariate tools meant to overlap the gathered information for a coherent understanding of the process and of the roles of each of the formulation components.

## 2. Materials and Methods

### 2.1. Materials

Microcrystalline cellulose and dicalcium phosphate were selected as diluents of this powder blend. Five sorts of microcrystalline cellulose (Vivapur 102, Vivapur 12, Vivapur 200, Vivapur 302 JRS Pharma, Rosenberg, Germany; Sigachi 102, Hyderabad, India) and four sorts of calcium hydrogen phosphate (anhydrous: Dicafos A60, Dicafos A150, JRS Pharma, Germany; dihydrate: Dicafos D160, Budenheim, Budenheim, Germany, Emcompress DC, JRS Pharma, Rosenberg, Germany) were included in the study. Sodium stearylfumarate (JRS Pharma, Rosenberg, Germany) and magnesium stearate (Union Derivan, Barcelona, Spain) were tested as lubricants, whereas sodium croscarmellose (JRS Pharma, Rosenberg, Germany) was chosen for its disintegrant properties. For the API, three different suppliers were included in the study. The identity of the API was not divulged due to confidentiality reasons.

### 2.2. Design of Experiments (DoE)

To understand the effect of the input factors on the tableting performance of the powder blends, a DoE approach was implemented in two stages. The screening design aimed to compare the tableting properties of different excipient combinations (placebo formulations). The sort of microcrystalline cellulose and the sort of calcium hydrogen phosphate were included as qualitative input variables. The two lubricants were used individually and combined (1:1 ratio), having this factor as a qualitative input variable with 3 levels of variation. The ratio of dicalcium phosphate was selected as a quantitative input variable, having a variation range between 10% and 70. The second quantitative input variable was the compression load (300 kg, 400 kg, 500 kg), corresponding to the standard output settings of the used tablet press.

Modde 12.1 optimization software (Sartorius, Umea, Sweden) was used to generate a D-Optimal design with 28 runs. The parameters obtained from compression analysis, namely the work of compression, in-die elastic recovery, tensile strength, detachment and ejection stress, were selected as relevant output variables for the optimization of this product.

The next stage of formulation development included a second D-optimal experimental design, with the API supplier and the redefined factors from the initial screening design.

All experiments designed under the DoE approach were performed using a randomized run order generated by the optimization software to minimize systematic bias and time-dependent effects. Model performance was evaluated with respect to the amount of captured variability (R2), predictive capacity (Q2), validity and reproducibility. ANOVA tests were used to evaluate the significance of the models and the lack of fit.

### 2.3. Compression Analysis

The tablets were obtained using a single-punch Gamlen GTP tablet press (Gamlen Tableting Ltd., Biocity Nottingham, UK). 100 mg-sized samples were compressed at three different loads (300, 400 and 500 kg) using ⌀6 mm flat-faced punches, respectively, with a compression and decompression speed of 10 mm/min. Force-displacement profiles were recorded with a 200 Hz graph sampling rate.

The compression analysis consisted of three different stages: compression, detachment and ejection. The work of compression was calculated as the area under the compression force-displacement curve using the areas of rectangles [36]. In-die elastic recovery was calculated indirectly according to the difference between the upper punch base position and the punch positions corresponding to the maximum (*t_max_*) and minimum (*t_0_*) value of the compression load [36,37].

(1) $$\mathrm{In-die}\;\mathrm{elastic}\;\mathrm{recovery}\;(\%)\;=\frac{t_{0}-t_{max}}{t_{max}}100$$

t_max_—punch position corresponding to the maximum compression load; t_0_—punch position corresponding to the minimum compression load.

From the detachment phase, the detachment stress was determined using the maximum force required to detach the tablet from the base of the die (or lower punch) divided by the contact surface area of the compact. The contact surface area was calculated as π × r^2^, where r is the radius of the tablet.

*Detachment Stress* (MPa) = *F*_D_/*S* (2)

F_D_—the maximum value of detachment force; S—contact surface area of the compact.

From the third and last stage, the ejection stress was calculated by using the maximum value of ejection force, tablet diameter and tablet thickness [37]. Tablet diameter and thickness were measured using a digital caliper (Yato, Poland).

(3) $$\mathrm{Ejection}\mathrm{stress}\;(\mathrm{MPa})\;=\frac{F_{E}}{\pi Dt}$$

F_E_—the maximum value of ejection force; D—tablet diameter; t—tablet thickness.

Tensile strength was calculated using the tablet breaking force, tablet diameter and π [37,38]

(4) $$\mathrm{Tensile}\;\mathrm{strength}\;(\mathrm{MPa})\;=\frac{2F_{B}}{\;\pi Dt}$$

F_B_—tablet breaking force; D—tablet diameter; t—tablet thickness.

### 2.4. Tablet Breaking Force

The breaking force of the tablets was measured after ejection using the Pharma Test, Germany hardness tester. Measurements were performed in triplicate.

### 2.5. Out of Die Compression Analysis Models

To further investigate the differences in the compression behavior of the raw materials, the compressibility, compactibility, and tabletability were evaluated using mathematical models.

The Heckel equation, reflecting the porosity—pressure relationship, was used to determine the mean yield pressure (Py) [8,39]

*ln* 1/*ε* = *k_H_P* + *A*; *Py* = 1/*k_H_* (5)

ε—porosity; P—compression pressure; k_H_—slope of the linear portion of the Heckel plot, A—intercept.

The Ryshkewitch–Duckworth equation, describing the TS—porosity relationship, was used to evaluate the interparticulate bonding capacity [8,40,41].

*TS* = *T*_0_ *exp* (−*k_b_ ε*) (6)

TS—tensile strength, T0—tensile strength at zero porosity; k_b_—bonding constant; ε—porosity.

The tabletability was estimated by fitting a power model on the TS versus compression pressure data, followed by the calculation of tabletability capacity (d) and pressure sensitivity index (g) [8].

*TS* = *dP^g^* (7)

TS—tensile strength; P—compression pressure; d—tabletability capacity; g—pressure sensitivity index.

The equation is derived from the Gurnham and Ryshkewitch Duckworth model:

(8) $$TS=\frac{T_{0}}{P_{0}^{\frac{k_{b}}{K}}}P^{\frac{k_{b}}{K}}$$

P_0_—pressure required to produce a zero porosity compact; T_0_—tensile strength of the compact with zero porosity; k_b_—bonding constant; P—the applied compression pressure; K—is related to the compressibility resistance of the powder

An increase in tabletability capacity (d), reflected by higher T_0_ and lower P_0_, suggests that a zero porosity compact can be obtained more easily by compressing the powder. A higher pressure sensitivity index (g) means that the powder is easier to transform into a tablet at higher pressure.

### 2.6. Scanning Electron Microscopy (SEM)

SEM micrographs were recorded on the diluents and API samples from different suppliers. Image acquisition was performed with a Quanta 3D FEG equipment (FEI, Thermo Scientific, Dreieich, Germany) at 15 kV after the samples were coated with Pd/Pt using an Automatic Sputter coater (Agar Scientific, Stansted, Essex, UK).

SEM images were analyzed using image analysis software (ImageJ software, National Institutes of Health, USA) to measure the particle length and width, followed by the calculation of minimum and maximum Feret diameters and the aspect ratio. A number-based particle size distribution was generated, and the particle size percentiles (d10, d50, d90) were determined by interpolation from the cumulative curve. Here, d10, d50, and d90 represent the particle diameters below which 10%, 50%, and 90% of the measured particles fall, respectively. In this respect, 100 particles were evaluated from 4 images, sampled from different regions of the sample.

### 2.7. Flowability

The bulk density was calculated as the ratio between the mass and the initial volume of 100 g sized samples loaded into a cylinder. The tapped density was calculated using the final volume obtained after a series of taps. The final volume was recorded after 750 and 1250 taps, whereas the recorded volume differed by more than 2 mL; another 500 taps were applied to reach the final volume. Tapping experiments were done using an Erweka SVM (Erweka, Langen, Germany) powder density tester.

Carr’s index and the Haussner ratio were calculated using the following equations [42]:

Carr’s index = (ρ_t_ − ρ_i_)/ρ_t_ × 100 (9)

Hausner ratio = ρ_t_/ρ_i_ (10)

ρ_t_—tapped density; ρ_i_—bulk density.

The angle of repose was determined by measuring the height of a cone-like pile of material formed by 50 g of powder that fell through a funnel from a height of 7.5 cm and with a funnel diameter of 10 mm. Experiments were done using a Powder flowability tester (Copley Scientific, Nottingham, UK).

### 2.8. Principal Component Analysis

PCA was used to compare the physical properties of different sorts of raw materials. Thus, the particle size and shape descriptors, flowability data, and parameters calculated from compaction analysis were initially scaled to unit variance. Separate models were developed for MCC, DCP and the API. Model interpretation was done by evaluating the amount of variability captured by the fitted latent variables and by generating Biplots.

## 3. Results and Discussions

### 3.1. Screening of Excipients

The importance of understanding the tableting properties of pharmaceutical excipients in the development of tablet formulations has been previously highlighted [22]. These tableting properties can be efficiently evaluated by studying the compressibility, compactibility and tabletability of the formulation under development. This way, products can be designed to undergo an efficient densification under compression (increased compressibility), to develop strong interparticulate bonds and large bonding surfaces (increased compactibility), and to exhibit a reduced friction with the die (ejection stress) to avoid tableting failures [22].

Considering all the effects involved in ensuring an optimal tableting performance, the screening study focused on evaluating different types/sorts of MCC and DCP, the ratio between the two diluents, and the type of lubricant.

The experimental runs were performed according to the D-optimal design presented in Table A1. Before proceeding to the interpretation of the obtained effects, the raw data were evaluated, and the models were optimized accordingly. Apart from ejection stress, all the models presented an optimal performance, explaining a large fraction of the response variation, having a good predictive capacity and validity. Replicate experiments performed at the defined design points were reproducible, showing minimal deviations with respect to the total variability recorded for the response (Table 1). Moreover, the model significance and absence of lack of fit was demonstrated using ANOVA (Table A3).

**Figure 1:**
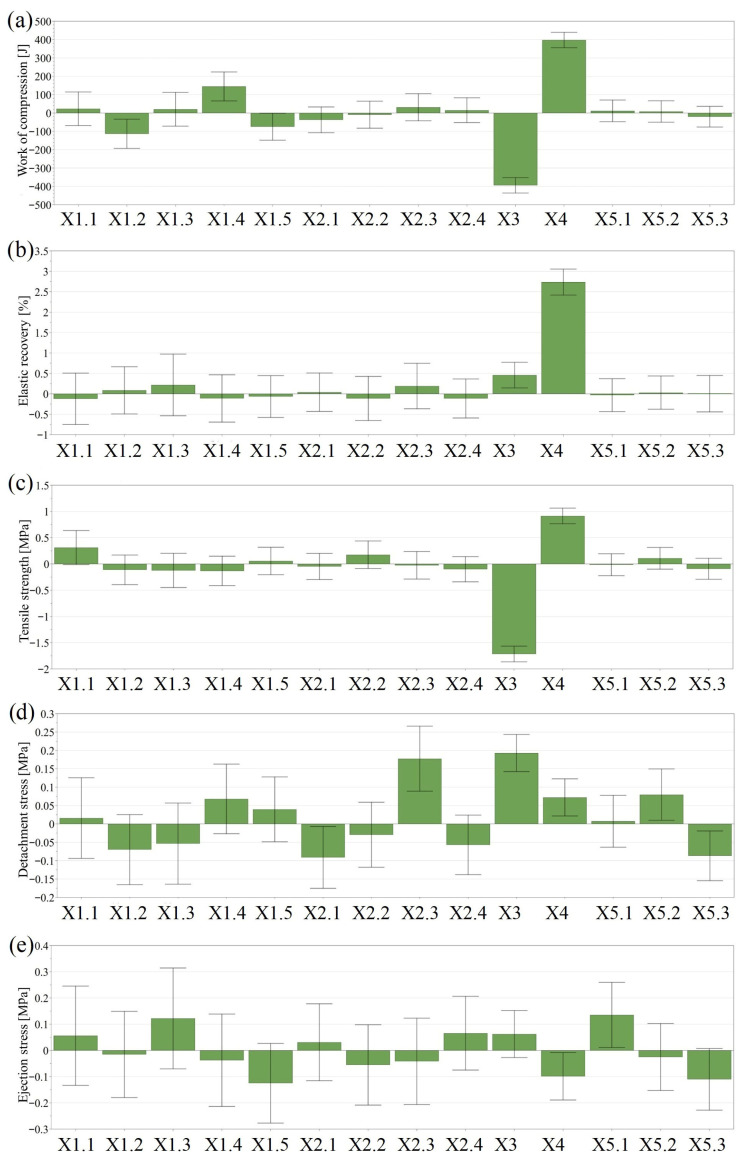
Coefficient plots presenting the influence of input variables on responses estimated using dynamic compaction analysis, in the screening study: (**a**) Work of compression; (**b**) In-die elastic recovery; (**c**) Tensile strength; (**d**) Detachment stress; (**e**) Ejection stress. X1.1: MCC-Vivapur 102; X1.2: MCC-Vivapur302; X1.3: MCC-Vivapur12; X1.4:Vivapur200; X1.5: MCC-Sigachi102; X2.1: DCP-Emcompress DC; X2.2: DCP-Dicafos A60; X2.3: DCP-Dicafos A150; X2.4: DCP-Dicafos D160; X3: DCP%; X4: Compression Load; X5.1: Lubricant-SSF; X5.2: Lubricant-MgST; X5.3: Lubricant-SSF:MgST.

The coefficient plots summarizing the effect of input variables on the evaluated responses are presented under Figure 1.

The consolidation during compaction, estimated through the work of compression as the sum of works involved in particle rearrangement, deformation and fragmentation, was influenced mostly by the compression force and the percentage of DCP, without any differences from different sorts of this diluent. On the other hand, blends prepared using VIVAPUR 302 and SIGACHI 102 types of MCC offered lower than average work of compression, while VIVAPUR 200 increased this response (Figure 1). The in-die elastic recovery of the powder blends increased up to 18.195%, being positively influenced by the applied compression force and the DCP content of the blend. An increase in the settings of these factors contributed to the reduction in the interparticulate bonding area after compaction.

The tensile strength increased with the increase in compression force and when the amount of DCP in the blend was reduced (Figure 1). Considering the different sorts of diluents, the use of VIVAPUR 102 was beneficial for obtaining a larger mechanical resistance.

For optimal manufacturing, the stress values associated with detachment and ejection should be lower than 3 MPa. The evaluated lubricants efficiently reduced stress values, providing values below the previously mentioned limit. Lower-than-average detachment and ejection stress values were obtained when MgST and SSF were used in combination. When used separately, the blends lubricated using MgST showed an above- average detachment stress, while SSF offered an above-average ejection stress. The detachment stress was positively influenced by the compression force and the percentage of DCP. Out of the DCP types, DICAFOS A150 increased, while EMCOMPRESS DC decreased this response.

The objective of the screening was to identify suitable sorts of excipients for the upcoming optimization step. The sort of MCC had a limited impact on the evaluated responses, although VIVAPUR 102 was beneficial for obtaining a larger TS. Thus, Sigachi 102 and VIVAPUR 102 types were selected to maintain the supplier of this diluent as a source of variation. In case of DCP, Emcompress DC and DICAFOS A60 were selected, as no undesired effect was detected for these sorts. As well as for the other diluent, the supplier-related variation was maintained.

The selection of the lubricant was the most straightforward, as the combined use of SSF and MgST was beneficial for both detachment and ejection stress reduction. The variation level of the DCP content was redefined due to its implication on the tablet’s TS. The initial variation level of 10–70% was reduced to 10–50%.

### 3.2. Optimization Study

The optimization study was performed with the redefined factors of the screening step, and added the API supplier as a new input variable. Adding the API supplier as a new input variable is scientifically justified because different suppliers often provide materials with inherent variability in critical raw material attributes (e.g., particle size and distribution, particle shape, density, flow properties, and other physicochemical characteristics). Therefore, product development can integrate this variability and systematically evaluate its impact on formulation performance. Working with alternative suppliers at this stage supports the development of robust formulations that can consistently deliver the desired product performance, despite normal differences in raw material attributes. This proactive approach strengthens process understanding, improves supply chain flexibility, and reduces the risk of performance variability during commercial manufacturing. The previously prepared placebo formulations were adjusted by adding 25% API and by proportionally reducing the other constituents. The selected API loading was selected to ensure dose feasibility, acceptable tablet size, and suitability for direct compression. The experimental runs were performed according to the D-optimal design presented in Table A2.

The predictive value of surrogate (placebo-based) systems for tableting behavior will depend on the API concentration and its physical properties influencing powder flow, densification, bond formation and elastic recovery. Low API concentration will ensure that the excipient matrix will govern the tableting behavior, whereas increasing API loading will deteriorate the quality of surrogate assumptions by changing the blend packing density, deformation behavior, and interparticle bonding.

Comparing the particle size descriptors of the APIs and excipients (Table 2), it can be anticipated that the finer and needle-like particles (high aspect ratio) will deteriorate the flowing properties, although the increased surface area could potentially lead to stronger bonding as a result of increased contact points. The size difference between API and excipients can also impact packing density and particle rearrangement during compression.

The work of compression of the API was between DCP and MCC; thus, the API containing blends were similar to placebo blends. The elastic recovery of the APIs was similar to MCC but lower compared to DCP. Therefore, the elastic recovery remained similar even after including 25% API in the blends.

The TS of pure API compacts could not be measured, suggesting poor tableting properties, even poorer than DCP, an excipient governed by a brittle deformation. The poorly bonding API slightly reduced the overall compact strength. However, the presence of plastically deforming MCC in the formulation maintains the TS above 2 MPa.

**Figure 2:**
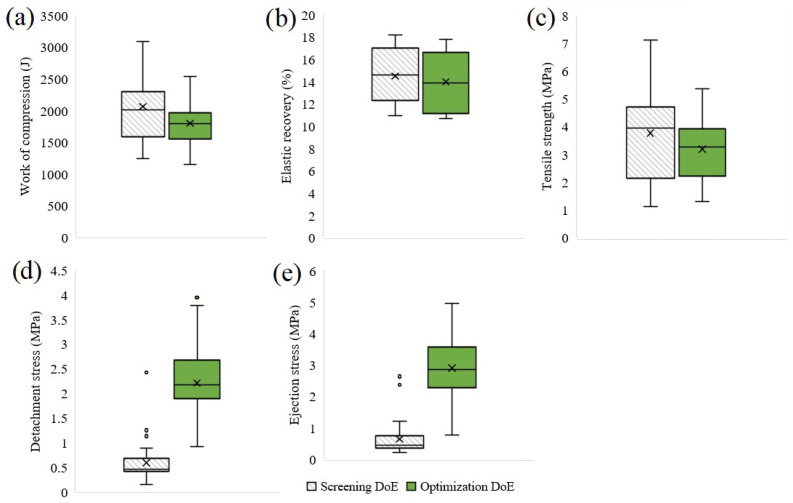
The effect of the API on the tableting properties of powder blends: (**a**) Work of compression; (**b**) In-die elastic recovery; (**c**) Tensile strength; (**d**) Detachment stress; (**e**) Ejection stress.

Including the API in the formulation had a major influence on the detachment and ejection of the tablets, making these processes more difficult (Figure 2). The DS of pure API was significantly higher compared to other formulation constituents, indicating possible sticking issues during tableting. In the case of ES, the API was situated between MCC and DCP, also contributing to the increased die wall friction.

**Figure 3:**
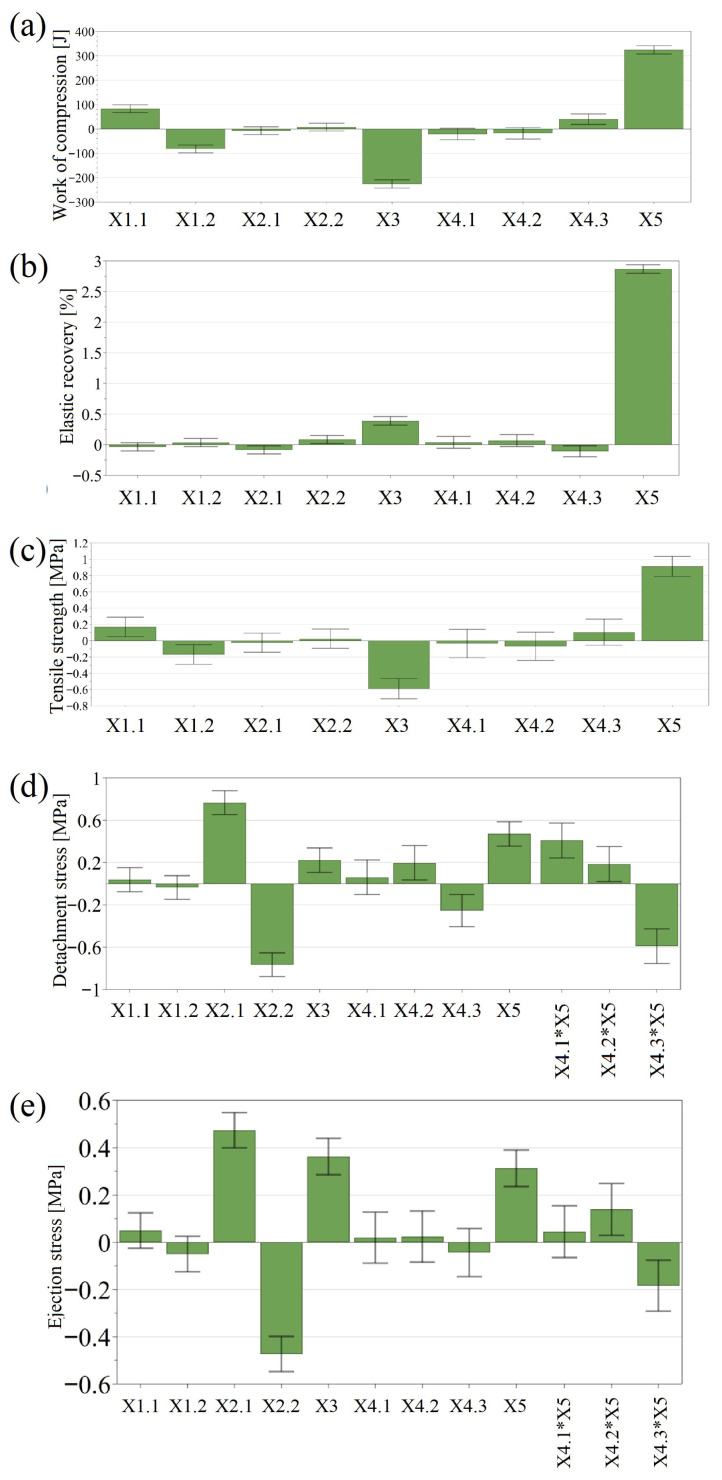
Coefficient plots presenting the influence of input variables on responses estimated using compression analysis, in the optimization study: (**a**) Work of compression; (**b**) In-die elastic recovery; (**c**) Tensile strength; (**d**) Detachment stress; (**e**) Ejection stress. X1.1: MCC-Vivapur102; X1.2: MCC-Sigachi102; X2.1: DCP-Emcompress DC; X2.2: DCP-DicafosA60; X3: DCP%; X4.1: API-sortA; X4.2: API-SortB; X4.3: API-SortC; X5: Compression load.

All the evaluated models were statistically significant (Table A3), explaining a large fraction of the response variation, having a good predictive capacity, validity and reproducibility (Table 1). The good reproducibility estimated from replicated experiments indicates a robust experimental procedure that can deliver consistent results, implying a reliable estimation of model coefficients. Model interpretation was performed using the coefficient plots presented in Figure 3.

The type of cellulose had an impact on the work of compression and tablet tensile strength. The higher work associated with VIVAPUR 102 offered above-average tensile strength. Although these effects are statistically significant, the practical relevance is reduced, as the mechanical resistance is predominantly influenced by the compression force and the DCP content of the blends. Therefore, the correct adjustments in the applied compression force can easily level out these differences.

The type of DCP did not influence the work of compression and TS, but was the main factor influencing the stress values from detachment and ejection. Formulations containing Emcompress DC were associated with larger stress values compared to DICAFOS A 60. On the other hand, the DCP content had a larger impact on the tablet ejection compared to the detachment of the tablet.

The factor related to the API supplier showed significant coefficients and interactions in the models developed for detachment and ejection stress.

**Figure 4:**
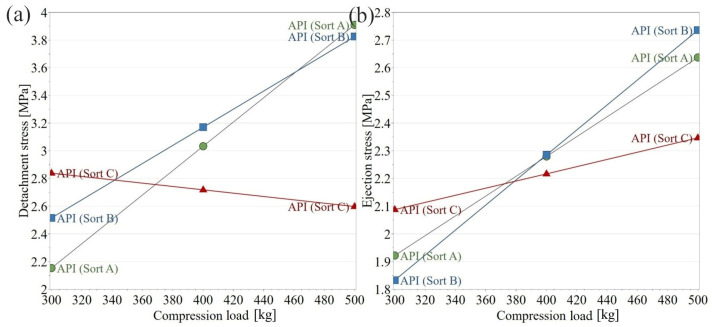
Interaction plot presenting the effect of API sort and compression force on the: (**a**) Detachment stress and (**b**) Ejection stress.

The interaction between the applied compression force and API sort showed that the change in detachment/ejection stress as a function of the applied compression force is dependent on the API sort. Sorts A and B exhibit an increasing tendency, while the detachment and ejection stress values for sort C changed at a lower rate (Figure 4).

### 3.3. Assessment of Tableting Properties and Root Cause Analysis

Osamura et al. presented a method that can be used to guide formulation development, offering an easy visualization of how the variation in formulation composition influences the tableting properties of the blends with respect to the TF and ES. This approach is beneficial for developing formulations with a lower risk of failure and enables a root cause analysis of failures with the possibility of identifying effective solutions [22]. Also, this approach was efficiently used to predict failures (capping and binding) at industrial scale [43].

The plot region is divided into four different regions in function of the ejection stress and compactibility of the investigated materials. Materials are considered to have appropriate tableting properties when the ES is below 5 MPa, and good compactibility when the TS reaches values above 2 MPa. Depending on how these properties combine, the tested materials can be found in region I (good compactibility and ejection stress); region II (good ejection stress, poor compactibility); region III (good compactibility and poor ejection stress) and region IV (poor ejection stress and compactibility).

**Figure 5:**
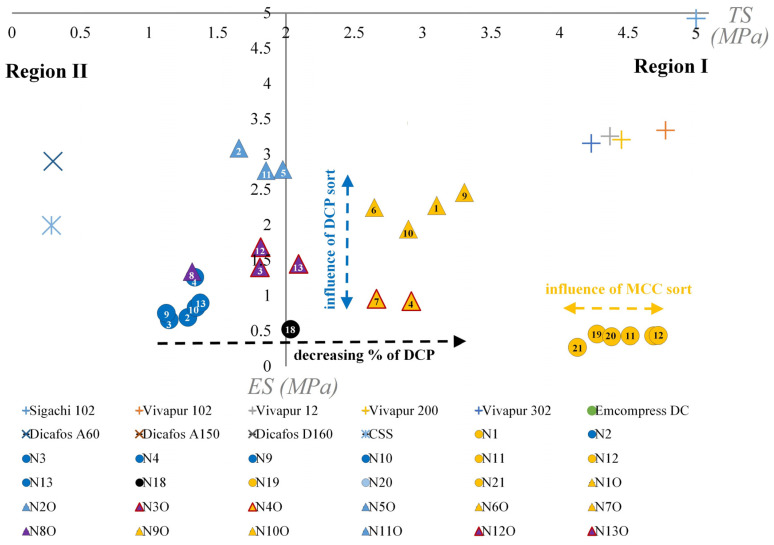
Tableting properties of raw materials and formulations prepared under the screening and optimization studies (300 kg compression force).

To provide an improved understanding of the effects involved, the raw materials were characterized individually by using the compaction simulator. The tableting properties of raw materials and of the blends prepared under the screening/optimization steps are presented in Figure 5.

Based on their positioning, the DCP sorts provided a poor TS, but were well differentiated with respect to ejection stress. DCP is a brittle material with a high fragmentation propensity that undergoes particle fragmentation even at low compression pressures [44,45]. The great extent of fragmentation ensures the division of the load on a larger area, therefore reducing the strength of the obtained bonds [44]. The influence of the initial particle size on the tabletability is reduced for highly fragmenting materials, as the contact surface area is increased [5,17]. This is the reason for the reduced differentiation of DCP sorts in the plot with respect to compactibility.

The reduced tabletability and predominant non-elastic deformation of DCP are also linked to high ejection forces due to an increased residual die wall stress [23]. Dicafos D160 (TS: 0.50 MPa; ES: 21.57 MPa), Dicafos A150 (TS: 0.49 MPa; ES: 14.18 MPa) and Emcompress DC (TS: 0.47 MPa; ES: 22.67 MPa) were hidden from the graph to increase the resolution on regions I and II.

MCC sorts provided a much higher TS, due to the plastic deformation of the excipient, with the sorts slightly scattered in the direction of the horizontal axis [17]. The irreversible modifications suffered by ductile materials (plastically deformable) offered an increased bonding area and tablet strength [4]. The ES values of MCC were below the 5 MPa limit, the larger stress values being associated with the Sigachi 102 type.

The lubrication of placebo mixtures from the screening design decreased the ejection stress, with all the formulations situated below the 5 MPa limit (independent of DCP sort). The clustering of placebo formulations was attributed to the DCP content of the blend. A higher percentage of DCP offered poor compactibility, whereas lower DCP content (higher MCC) significantly increased the TS of the prepared compacts. Just as for the pure MCC types, these blends were also slightly scattered in the direction of the horizontal axis. In case of the placebo formulations, just as for the pure MCC sorts, the Sigachi 102 and Vivapur 102-based blends were positioned towards higher TS values.

The inclusion of the API into the formulation led to the reduction in tablet TS, mainly due to the lower compactibility of this component and due to its relatively high amount (25%). The API sorts could not be plotted individually as the hardness of the prepared compacts was below the measurable range. Interestingly, the inclusion of the API into the blend created a separation of formulations in function of the DCP sort. For both high and low percentages of DCP, blends prepared using Emcompress DC were separated from Dicafos A60 and positioned towards higher values on the ejection stress scale. These effects were not visible in the absence of the API (placebo formulations), where multiple sorts of DCP were screened.

For an extended root cause analysis of the observed effects, separate PCA models were developed using the physical properties of MCC, DCP, and the API.

**Figure 6:**
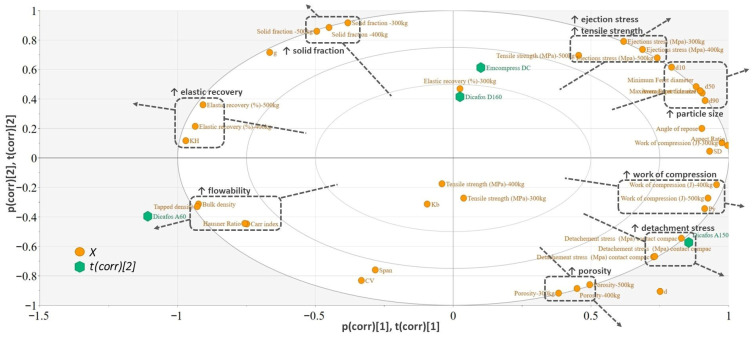
Biplot of the PCA model built for DCP.

The biplot of the PCA model built for DCP is presented in Figure 6. Biplots simultaneously represent scores and loading, enabling an easy visualization of the differences between observations and of the correlations between variables. Observations (excipients) situated near a variable are high in this variable, and low in the variable found in the opposite direction.

Based on the placement of the studied physical characteristics, a larger particle size for DCP will increase the work of compression (due to the extensive fragmentation), the compact tensile strength and stress values associated with tablet ejections. The higher tensile strength is also a result of the lower in-die elastic recovery. Also, the flowability will be improved (lower values for the Carr index and Haussner ratio), hence the negative correlation with this group of variables.

**Figure A1:**
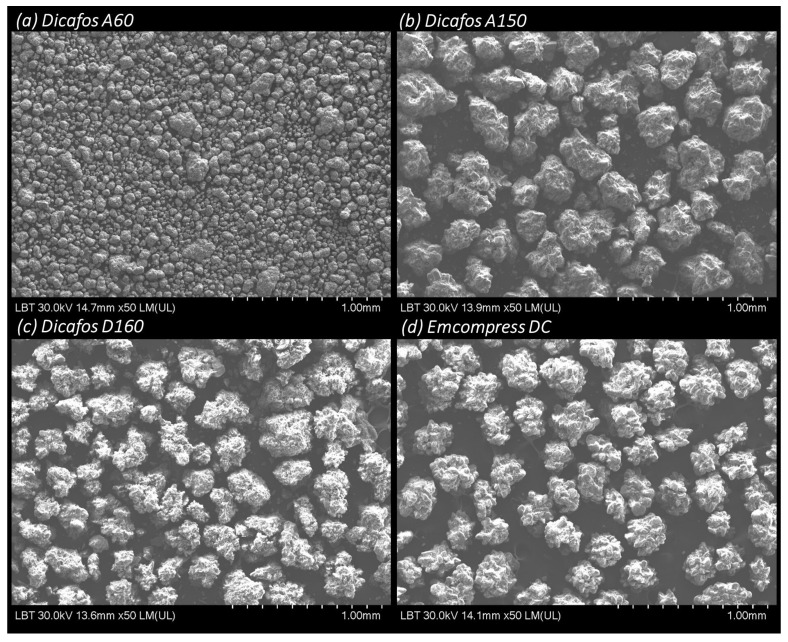
SEM micrographs of DCP sorts included in the study (**a**) Dicafos A60; (**b**) Dicafos A150; (**c**) Dicafos D160; (**d**) Emcompress DC.

The main physical difference between Emcompress DC and Dicafos A60 types was related to the particles of these sorts (Figure A1). Despite the smaller particles of Dicafos A60 reducing the compactibility of the pure excipient, this effect was not observed when it was included in a blend. However, a small separation with respect to ejection stress could be observed only when the API was also included in the formulation (Figure 5). Including in the blend another component with high ejection stress reduced the ability of the lubricant to efficiently cover the surface of these particles. Therefore, the previously leveled differences between DCP sorts in the placebo formulations are highlighted to a smaller extent. During compaction, the larger particles of DCP generated more non-lubricated surfaces upon fragmentation, which eventually affected the ejection stress.

Dicafos A150 is also a sort with an increased particle size, but has a larger detachment stress compared to Emcompress DC. This effect was also highlighted in the case of the screening stage, confirming the additive nature of these properties and the utility of using the properties of individual components to estimate the behavior of their blend.

Larger particle size was associated with a more pronounced brittle fracture, as suggested by the large mean yield pressure (Py > 400 MPa). Moreover, these excipients were classified as difficult to compact based on the bonding capacity (Kb > 10).

**Figure 7:**
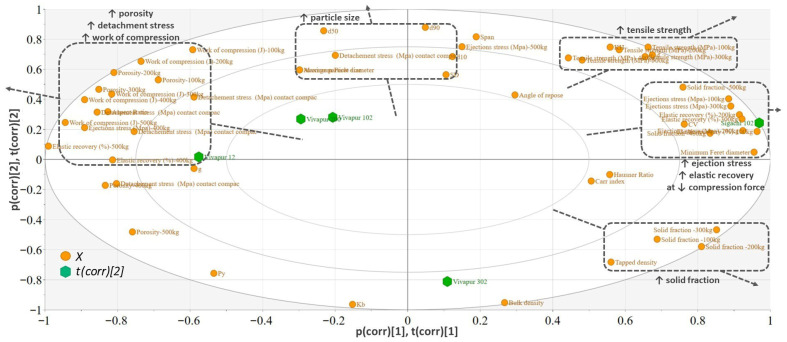
Biplot of the PCA model built for MCC.

The biplot generated to highlight the differences between MCC sorts is presented in Figure 7. The MCC sorts are more tightly clustered in the ‘tableting properties’ plot compared to DCP, although a small differentiation can be observed with respect to compactibility (Figure 5).

TS was positively correlated with the level of plasticity in the characterized material, estimated through the k_H_ and Py parameters. A high k_H_/low Py suggests an easier and more rapid plastic deformation that creates sufficient contact area between the particles. The tabletability capacity parameter (d) estimated using the power model was another parameter well correlated with the TS of compacts prepared from pure MCC samples. A larger tabletability capacity was responsible for obtaining high TS even at lower pressures [8,39].

**Figure A2:**
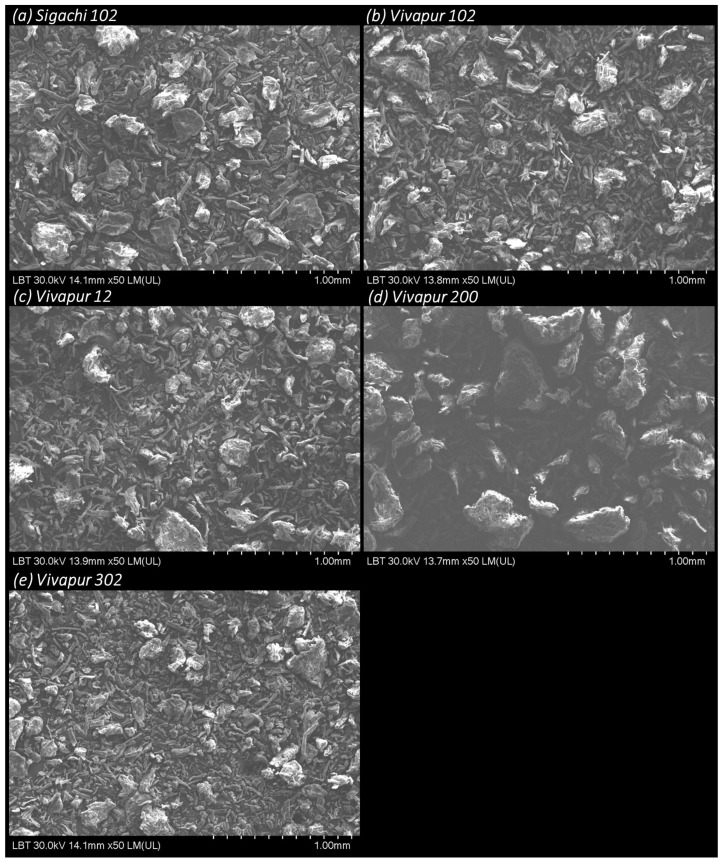
SEM micrographs of MCC sorts included in the study (**a**)—Sigachi 102; (**b**)—Vivapur 102; (**c**)—Vivapur 12; (**d**)—Vivapur 200; (**e**)—Vivapur 302.

Sigachi 102 presented the highest k_H_ (0.011)/lowest Py (90.7) and the largest tabletability capacity (0.0251) out of the MCC types. Thus, it delivered the hardest compact when it was compressed individually (Table 2). This excipient also presented an increased ejection stress, and the particle had a larger minimum Ferret diameter (60 µm) compared to other sorts (average ± standard deviation: 50 µm ± 1 µm) (Figure A2).

Regarding the compactibility of MCC sorts, the k_b_ parameter ranged between 6.33 and 7.45, suggesting easily compactable materials according to the classification system proposed by Dai et al. [8]. Vivapur 302 was the most difficult to compact type of MCC.

Only for MCC, it was possible to model the compression profile and determine these parameters, as the prepared compacts were in a measurable range of mechanical resistance.

The biplot also identified a strong correlation between the work of compression porosity and detachment stress for this diluent.

**Figure 8:**
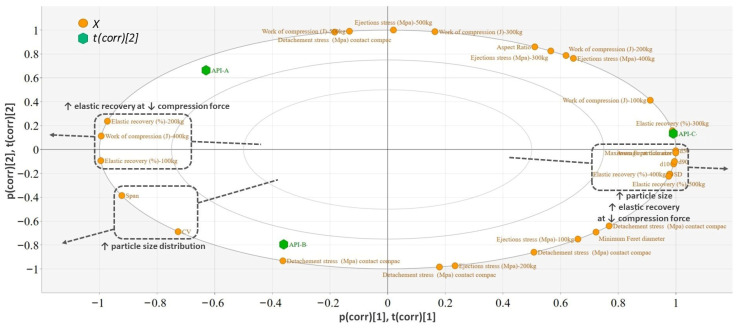
Biplot of the PCA model built for the API.

**Figure 9:**
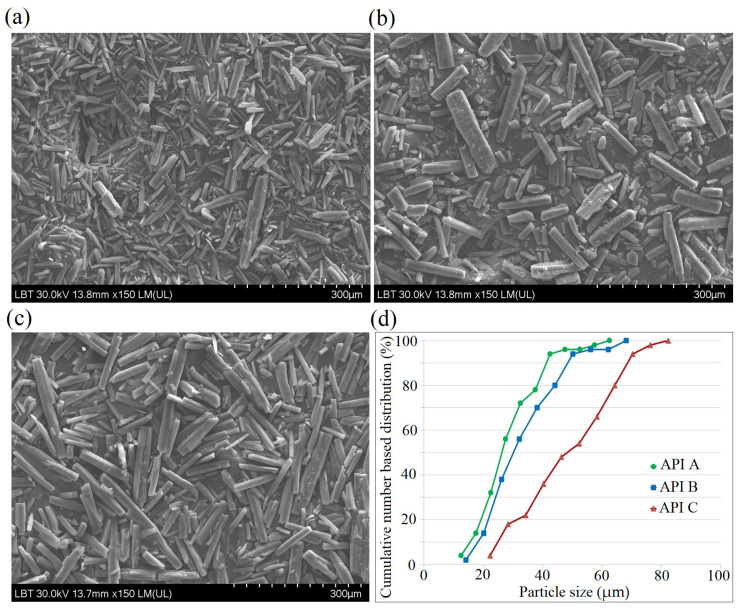
SEM micrographs of API particles originating from different suppliers: (**a**) API A; (**b**) API B; (**c**) API C, and (**d**) the cumulative frequency of particle size.

The biplot of the PCA model built on the physical characteristics and tableting parameters of the API sorts revealed the in-die elastic recovery, particle size and particle distribution as the main discriminatory variables between sorts (Figure 8). Sort C had a coarser particle size (d10, d50, d90) compared to sorts A and B (Figure 9). The coarser particle size resulted in lower elastic recovery and lower compression forces, whereas higher tableting forces led to an increase in elastic recovery. From a practical point of view, the size of these differences was not considered practically relevant as they were not greater than 2%. At 500 kg, the recorded elastic recovery was 14.1% for Api A, 14.6% for API B, and API C: 15.6%.

The detachment and ejection stress of the pure API powders presented a limited variation between the investigated suppliers, without any noticeable trends. When the API was included in the formulation, an interaction was highlighted with the compression force, related to the evolution of detachment and ejection stress values. The distinct evolution of detachment and ejection stress observed for blends containing APIs of different particle sizes is unlikely to originate from differences in bulk elastic recovery, as the in-die elastic recovery remained comparable (≈17%) irrespective of API particle size. Given that residual radial stress is primarily governed by the elastic strain retained during compression and released upon unloading, the similar recovery values suggest that the magnitude and pressure-dependence of residual die wall stress are broadly comparable between formulations with different API suppliers. Moreover, the absence of significant differences in the ejection behavior of the neat APIs further indicates that intrinsic material properties are not the dominant factor. Instead, the differences observed in the multicomponent blends likely arise from the die-wall friction. The finer API (d50 ≈ 30 µm), characterized by a higher specific surface area, may influence lubricant distribution and surface coverage efficiency within the blend. At a fixed lubricant concentration, increased surface area can reduce the availability of lubricant at the die wall interface, particularly at higher compaction pressures where particle rearrangement and surface renewal occur. This would result in a stronger pressure-dependent increase in the effective die-wall friction coefficient, thereby producing a steeper rise in ejection stress (Figure 4). In contrast, the coarser API (d50 ≈ 50 µm) would impose a lower surface area demand on the lubricant system, potentially allowing more stable die-wall lubrication and a correspondingly weaker dependence of ejection stress on compression force. The present findings suggest that particle-size–mediated modulation of interfacial lubrication, rather than differences in residual die wall stress, governs the observed ejection behavior. Furthermore, lubrication is known to reduce ejection force primarily by lowering the die-wall friction coefficient, while being considerably less effective when high ejection forces originate from elevated residual radial stress [23].

This interpretation is consistent with previous reports highlighting the influence of particle size on interfacial friction phenomena. Abdel-Hamid et al. reported an increased tendency for friction and sticking with decreasing granule size, which was attributed to enhanced interaction with the die wall [46]. More recently, Casian et al. demonstrated that the particle size distribution of formulation components can be predictive of detachment and ejection stress behavior [47]. Excessive friction at the tablet–die wall interface restricts axial expansion of the compact near the die surface during decompression, while the inner regions of the tablet experience comparatively less constraint. This non-uniform stress distribution may promote microcrack formation and, under more severe conditions, lead to macroscopic defects such as capping or lamination [23].

## 4. Conclusions

The current study presents a sequential strategy for the development of directly compressed tablets using QbD tools. Two DoE stages were designed where placebo and drug-containing formulations were assessed through compression analysis. Further, MVDA was meant to develop models that correlate the compaction properties of placebo formulations with those of API loaded formulations and the physical properties of individual excipients and APIs.

First, the DoE for screening purposes included several sorts of the two selected diluents, MCC and DCP, the diluent ratio, the compaction force, and the lubricant type as input variables. It revealed statistically significant effects of the DCP ratio and sort, compaction force, and lubricant type on the compaction parameters, which guided the choice of inputs in the following optimization DoE. The screening DoE provided an overview of the compression behavior of placebo powder blends containing both ductile and brittle materials. As the resulting formulations could accommodate different APIs with particular compressibility and compactibility profiles, it may be regarded as a versatile tool in the further development of directly compressible products. This hypothesis was tested by the inclusion of three sorts of API in the screening-derived optimization design. As expected, the API had a negative impact on compactibility, but only two of the tested sorts increased detachments and ejection stresses while compression forces increased.

Finally, MVDA correlated the DoE data, while the root cause analysis explained the relations between microscopic particle properties, such as particle sizes and bulk properties, and in-die and out-of-die compression features.

## Figures and Tables

**Table 1:** Performance parameters of PLS/MLR models developed under the screening and optimization stages.

Model Performance Parameters	R^2^	Q^2^	Validity	Reproducibility
**Screening study**				
Work of compression	0.982	0.945	0.784	0.981
In-die elastic recovery	0.968	0.925	0.927	0.916
Tensile strength	0.980	0.930	0.522	0.993
Detachment stress	0.873	0.576	0.617	0.941
Ejection stress	0.562	−0.422	0.711	0.677
**Optimization study**				
Work of compression	0.995	0.939	0.435	0.998
In-die elastic recovery	0.998	0.983	0.278	0.999
Tensile strength	0.961	0.827	0.737	0.956
Detachment stress	0.969	0.829	0.090	0.998
Ejection stress	0.981	0.884	0.757	0.968

**Table 2:** Physical properties of API types in relation to excipient properties.

**Particle Size Descriptors**	**d10**	**d50**	**d90**	**Minimum Feret Diameter**	**Maximum Feret Diameter**	**Aspect Ratio**
Sigachi 102	154	227	360	59	240	4.14
Vivapur 102	152	228	342	52	237	4.70
Vivapur 12	140	226	331	50	265	5.62
Vivapur 200	166	250	388	49	231	4.92
Vivapur 302	139	199	286	52	209	4.19
Emcompress DC	428	543	645	409	543	1.37
Dicafos A60	22	32	47	27	34	1.26
Dicafos A150	326	541	719	393	537	1.42
Dicafos D160	343	499	691	387	508	1.36
API-A	16	26	41	5	28	5.28
API-B	18	30	48	8	32	3.93
API-C	25	48	69	9	48	5.58
**Tableting Properties**	**Work of Compression (J)** **—** **500 kg**	**Elastic Recovery (%)** **—** **500 kg**	**Tensile Strength (MPa)** **—** **500 kg**	**Detachment Stress (Mpa)** **—** **500 kg**	**Ejections Stress (Mpa)** **—** **500 kg**	**Solid Fraction** **—** **500 kg**
Sigachi 102	2965	13.773	7.897	6.945	4.150	0.925
Vivapur 102	3371	14.130	7.899	5.580	4.092	0.909
Vivapur 12	3384	14.298	7.235	7.708	4.040	0.903
Vivapur 200	3437	14.198	7.147	7.808	4.232	0.917
Vivapur 302	3159	14.007	6.970	4.469	3.993	0.906
Emcompress DC	1445	18.745	0.673	5.471	39.912	0.756
Dicafos A60	1297	19.541	0.450	5.578	13.006	0.698
Dicafos A150	1825	16.139	0.602	9.697	32.451	0.577
Dicafos D160	1580	18.043	0.859	5.763	38.362	0.738
API-A	2427	14.102	-	20.369	18.261	-
API-B	1929	14.672	-	17.640	15.266	-
API-C	2188	15.617	-	18.936	17.225	-

## Data Availability

The raw data supporting the conclusions of this article will be made available by the authors on request.

## Abbreviations

The following abbreviations are used in this manuscript:

A	intercept of the Heckel plot
ANOVA	analysis of variance
API	active pharmaceutical ingredient
D	tablet diameter
DCP	dicalcium phosphate
DoE	design of experiments
DS	detachment stress
d	tabletability capacity
ES	ejection stress
F_B_	tablet breaking force
F_D_	the maximum value of detachment force
F_E_	the maximum value of ejection force
g	pressure sensitivity index
K	compressibility resistance of the powder
k_b_	bonding constant Ryshkewitch-Duckworth equation
k_H_	slope of the linear portion of the Heckel plot
MCC	microcrystalline cellulose
MgST	magnesium stearate
MLR	multiple linear regression
MVDA	multivariate data analysis
P_0_	pressure required to produce a zero porosity compact
PCA	principal component analysis
PLS	partial least squares
Py	mean yield pressure
Q^2^	predictive capacity
QbD	quality by design
R^2^	the amount of captured variability
S	contact surface area of the compact
SEM	scanning electron microscopy
SSF	sodium stearyl fumarate
t	tablet thickness
t_0_	base punch position corresponding to the minimum compression load
T_0_	tensile strength at zero porosity
t_max_	base punch position corresponding to the maximum compression load
TS	tensile strength
ε	powder bed porosity
ρ_i_	bulk density
ρ_t_	tapped density

## Appendix A

**Table A1:** Formulations prepared in the screening study.

Exp.	R.O	MCC Sort	DCP Sort	DCP%	Compression Load [kg]	Lubricant Type	Work of Compression [J]	In Die-Elastic Recovery [%]	Tensile Strength [MPa]	Detachment Stress [MPa]	Ejection Stress [MPa]
N1	23	SIGACHI 102	EMCOMP DC	10	300	SSF	1910.48	11.288	4.685	0.441907	0.432
N2	11	SIGACHI 102	EMCOMP DC	70	300	SSF	1285.96	12.432	1.284	0.506847	0.690
N3	20	VIVAPUR 302	DI-CAFOS A60	70	300	SSF	1241.61	12.460	1.148	0.524597	0.655
N4	7	VIVAPUR 200	DI-CAFOS A150	70	300	SSF	1488.27	12.195	1.336	1.2212	1.258
N5	13	VIVAPUR 200	DI-CAFOS A60	10	500	SSF	3085.98	16.681	6.533	0.386771	0.447
N6	15	VIVAPUR 102	DI-CAFOS A150	10	500	SSF	3014.24	17.090	7.116	0.469445	0.495
N7	6	VIVAPUR 302	DI-CAFOS D160	10	500	SSF	2825.06	16.701	6.195	0.361781	0.430
N8	22	VIVAPUR 12	DI-CAFOS D160	70	500	SSF	2031.64	18.152	2.453	0.835841	1.133
N9	28	VIVAPUR 302	EMCOMP DC	70	300	MgST	1259.03	12.576	1.127	0.515368	0.753
N10	25	VIVAPUR 12	EMCOMP DC	70	300	MgST	1305.09	12.467	1.344	0.528097	0.832
N11	1	SIGACHI 102	DI-CAFOS A60	10	300	MgST	1947.75	11.043	4.513	0.302545	0.432
N12	16	VIVAPUR 102	DI-CAFOS D160	10	300	MgST	2184.22	10.977	4.718	0.341269	0.437
N13	19	VIVAPUR 200	DI-CAFOS D160	70	300	MgST	1435.47	12.295	1.375	0.764017	0.895
N14	14	VIVAPUR 302	EMCOMP DC	10	500	MgST	2753.14	17.370	6.973	0.387216	0.491
N15	18	VIVAPUR 12	DI-CAFOS A60	10	500	MgST	2936.39	17.072	6.970	0.347247	0.422
N16	8	VIVAPUR 200	DI-CAFOS A150	70	500	MgST	2216.92	18.195	2.959	2.37261	2.425
N17	12	SIGACHI 102	DI-CAFOS A150	70	500	MgST	2124.68	18.169	2.945	2.6344	0.168
N18	4	VIVAPUR 102	DI-CAFOS A60	70	300	SSF:MgST	1305.61	12.319	2.035	0.781555	0.523
N19	9	VIVAPUR 302	DI-CAFOS A150	10	300	SSF:MgST	1879.54	11.504	4.272	0.22365	0.456
N20	27	VIVAPUR 12	DI-CAFOS A150	10	300	SSF:MgST	2234.5	11.200	4.377	0.380221	0.419
N21	3	VIVAPUR 200	DI-CAFOS D160	10	300	SSF:MgST	2315.8	12.706	4.129	0.310815	0.267
N22	17	VIVAPUR 102	EMCOMP DC	70	500	SSF:MgST	1985.69	17.802	2.815	0.601961	0.659
N23	21	VIVAPUR 200	EMCOMP DC	10	500	SSF:MgST	2998.25	16.792	6.039	0.284999	0.182
N24	2	VIVAPUR 302	DI-CAFOS A60	70	500	SSF:MgST	1940.35	15.614	3.035	0.763123	0.221
N25	26	SIGACHI 102	DI-CAFOS D160	70	500	SSF:MgST	2218.39	16.123	2.880	0.761177	0.154
N26	10	SIGACHI 102	DI-CAFOS D160	40	400	SSF:MgST	2022.89	14.666	4.123	0.307887	0.369
N27	24	SIGACHI 102	DI-CAFOS D160	40	400	SSF:MgST	1886.49	15.942	3.844	0.407098	0.621
N28	5	SIGACHI 102	DI-CAFOS D160	40	400	SSF:MgST	2015.28	14.578	4.074	0.372877	0.630

R.O.—run order.

**Table A2:** Formulations prepared in the optimization study.

Exp.	R.O	MCC Sort	DCP Sort	DCP%	API Sort	Compression Load [kg]	Work of Compression [J]	Elastic Recovery [%]	Tensile Strength [MPa}	Detachment Stress [MPa]	Ejection Stress [MPa]
N1	22	VIVAPUR 102	EMCOMP DC	10	Sort A	300	1743.76	10.942	3.100	2.709	2.275
N2	11	SIGACHI 102	EMCOMP DC	50	Sort A	300	1195.06	11.332	1.657	3.650	3.088
N3	20	VIVAPUR 102	DI-CAFOS D160	50	Sort A	300	1342.08	11.660	1.809	1.488	1.401
N4	26	SIGACHI 102	DI-CAFOS D160	10	Sort A	300	1643.03	11.098	2.915	0.764	0.923
N5	12	VIVAPUR 102	EMCOMP DC	50	Sort B	300	1336.1	11.049	1.977	4.114	2.786
N6	17	SIGACHI 102	EMCOMP DC	10	Sort B	300	1604.46	10.678	2.646	3.265	2.247
N7	15	VIVAPUR 102	DI-CAFOS D160	10	Sort B	300	1726.04	11.255	2.661	1.273	0.954
N8	23	SIGACHI 102	DI-CAFOS D160	50	Sort B	300	1157.42	12.250	1.315	1.411	1.341
N9	14	VIVAPUR 102	EMCOMP DC	10	Sort C	300	1834.11	10.846	3.303	3.365	2.460
N10	27	SIGACHI 102	EMCOMP DC	10	Sort C	300	1632	10.972	2.894	3.190	1.942
N11	7	SIGACHI 102	EMCOMP DC	50	Sort C	300	1285.37	11.283	1.855	3.424	2.770
N12	25	VIVAPUR 102	DI-CAFOS D160	50	Sort C	300	1382.04	11.438	1.815	1.967	1.687
N13	2	SIGACHI 102	DI-CAFOS D160	10	Sort C	300	1542.26	11.059	2.092	2.377	1.451
N14	4	VIVAPUR 102	EMCOMP DC	50	Sort A	500	1890.2	17.453	3.434	4.739	3.945
N15	3	SIGACHI 102	EMCOMP DC	10	Sort A	500	2226.51	16.464	4.446	4.446	2.212
N16	6	VIVAPUR 102	DI-CAFOS D160	10	Sort A	500	2492.65	16.516	4.705	2.989	1.936
N17	21	SIGACHI 102	DI-CAFOS D160	50	Sort A	500	1776.84	17.688	3.327	3.467	2.455
N18	8	VIVAPUR 102	EMCOMP DC	10	Sort B	500	2484.22	16.668	5.166	4.292	2.534
N19	28	SIGACHI 102	EMCOMP DC	50	Sort B	500	1784.98	17.727	3.033	4.953	3.779
N20	13	VIVAPUR 102	DI-CAFOS D160	50	Sort B	500	1971.75	17.449	3.993	3.310	2.717
N21	18	SIGACHI 102	DI-CAFOS D160	10	Sort B	500	2270.11	16.316	4.338	2.741	1.913
N22	9	VIVAPUR 102	EMCOMP DC	50	Sort C	500	1971.86	17.141	3.228	3.610	3.032
N23	16	SIGACHI 102	EMCOMP DC	10	Sort C	500	2294.91	16.361	4.423	2.317	1.894
N24	1	VIVAPUR 102	DI-CAFOS D160	10	Sort C	500	2535.73	16.425	5.357	2.259	1.989
N25	10	SIGACHI 102	DI-CAFOS D160	50	Sort C	500	1862.1	17.808	3.537	1.962	2.262
N26	24	SIGACHI 102	DI-CAFOS D160	30	Sort C	400	1802.86	13.852	3.343	2.352	1.997
N27	19	SIGACHI 102	DI-CAFOS D160	30	Sort C	400	1799.29	13.951	3.744	2.351	2.146
N28	5	SIGACHI 102	DI-CAFOS D160	30	Sort C	400	1829.3	13.885	3.368	2.434	1.891

R.O.—run order.

**Table A3:** ANOVA results for model significance and lack of fit.

	Regression	Lack of Fit
**Screening study**		
Work of compression	7.67 × 10^−12^	4.23 × 10^−1^
Elastic recovery	6.18 × 10^−8^	7.50 × 10^−1^
Tensile strength	1.70 × 10^−11^	1.48 × 10^−1^
Detachment stress	3.12 × 10^−5^	2.17 × 10^−1^
Ejection stress	1.54 × 10^−1^	3.16 × 10^−1^
**Optimization study**		
Work of compression	1.74 × 10^−8^	1.05 × 10^−1^
Elastic recovery	1.42 × 10^−10^	5.59 × 10^−2^
Tensile strength	3.55 × 10^−6^	3.50 × 10^−1^
Detachment stress	1.45 × 10^−7^	2.64 × 10^−2^
Ejection stress	1.06 × 10^−5^	3.80 × 10^−1^
